# Rupture of Splenic Artery Aneurysm in Patient with ACTN2 Mutation

**DOI:** 10.3390/jcm12144729

**Published:** 2023-07-17

**Authors:** Martina Palughi, Pasqualino Sirignano, Nazzareno Stella, Michele Rossi, Laura Fiorani, Maurizio Taurino

**Affiliations:** 1 Vascular and Endovascular Surgery Unit, Sant’Andrea Hospital of Rome, Department of Clinical and Molecular Medicine, “Sapienza” University of Rome, 00189 Rome, Italy; 2 Vascular and Endovascular Surgery Unit, Sant’Andrea Hospital of Rome, Department of General and Specialistic Surgery, “Sapienza” University of Rome, 00189 Rome, Italy; 3 Interventional Radiology Unit, Sant’Andrea Hospital of Rome, Department Medical-Surgical Sciences and Translational Medicine, “Sapienza” University of Rome, 00189 Rome, Italy; 4 Cariology Unit, Sant’Andrea Hospital of Rome, 00189 Rome, Italy

**Keywords:** splenic artery aneurysm, ACTN2 mutation, coil embolization, congenital disease

## Abstract

Here, we report a case of splenic artery aneurysm rupture in a patient with known heterozygosity mutation of the ACTN2 gene (variant *c.971G > A p.Arg324Gln*). The patient came to our emergency department with epigastric pain radiating to the lumbar area, with an absence of peritonism signs. An abdominal computed tomography angiography showed a ruptured huge (5 cm) splenic artery aneurysm. Therefore, the patient underwent emergency endovascular coil embolization with complete aneurysm exclusion. The postoperative course was uneventful, until postoperative day five when the patient developed a symptomatic supraventricular tachycardia in the absence of echocardiographic alterations. The signs and symptoms disappeared after three days of medical management. The patient was discharged on the 14th postoperative day in good clinical condition under verapamil and anti-platelet therapy. Although ACTN2 mutation was associated with cardiac and peripheral vascular disease occurrence, to the best of our knowledge, the present case is the first report of a visceral (splenic) aneurysm directly linked with this rare mutation.

## 1. Introduction

The actinins are a multigene family of four actin-binding proteins, members of the spectrin family, that cross-link actin filaments [1]. Among them, α-actin 2 (a protein encoded by the ACTN2 gene) is a component of the cytoskeleton of cardiac, vascular, and skeletal muscle cells. Located in the z-disc of the sarcomere, the protein creates a connection between antiparallel actin filaments, and it gives stability to the sarcomere through the bonds to the n-terminal titins [2,3].

The functions of α-actin 2 are complex: it promotes protein binding within the sarcomere, regulates calcium, potassium and sodium ion channels, and promotes the transactivation of nuclear receptors in order to control the expression of smooth muscle cell (SMC) contractile genes [4,5,6,7]. Forty-nine rare variants of ACTN2 have been identified through three different genetic sequencing projects [8], and linked with cardiac diseases, such as cardiac arrhythmias, hypertrophic cardiomyopathy, ischemic stroke, and aortic aneurysm [9]. Predominantly, ACTN2 mutations are well-known causes of familiar thoracic aorta aneurismatic diseases, but never medium and small artery aneurysmal degeneration [9].

Here, we present a case of splenic artery aneurysm rupture in a patient with heterozygous mutation of the ACTN2 gene (variant *c.971G > A p.Arg324Gln*). The patient provided written informed consent for the report of his case details and imaging studies.

## 2. Detailed Case Description

A 39-year-old male came to the emergency room for epigastric pain radiating to the lumbar area, with an absence of peritonism signs. The patient was hemodynamically stable, with systemic blood pressure at 110/60 mmhg, heart rate at 100 beats/min, and hemoglobin at 13 g/dL. 

His medical history included a previous diagnosis of Gilbert’s disease and a heterozygosity mutation of ACTN2 (variant c*.971G > A p.Arg324Gln*). The patient never had any symptoms but underwent genetic tests because his son was diagnosed with non-compact myocardial syndrome.

**Figure 1:**
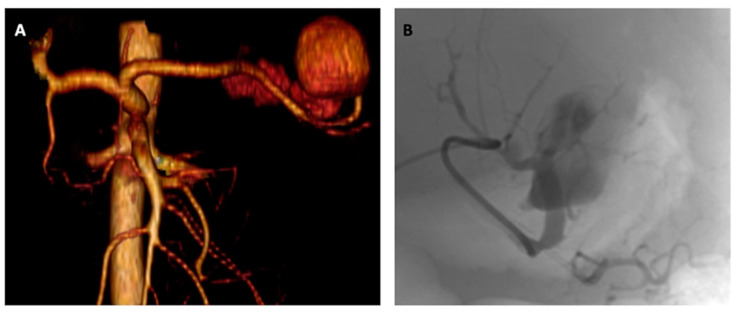
(**A**) Three-dimensional volume rendering CTA reconstruction showing the huge ruptured splenic artery aneurysm, and (**B**) intraoperative super-selective digital subtraction angiography confirming the CTA findings.

The patient underwent a computed tomography angiography (CTA) showing a huge (5 cm) aneurysmatic dilatation of the distal splenic artery portion with active intraperitoneal contrast medium extravasation (Figure 1). 

**Figure 2:**
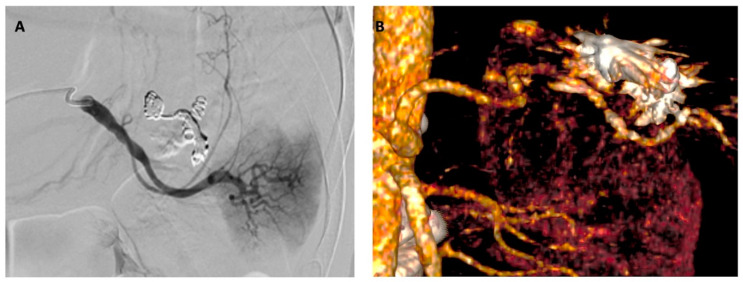
(**A**) Completion angiography showing complete splenic artery exclusion after coil embolization, and (**B**) postoperative three-dimensional volume rendering CTA reconstruction confirming the complete aneurysm exclusion.

Consequently, an emergent coil embolization was carried out. The procedure was performed under local anesthesia and by percutaneous common right femoral artery access. Splenic artery aneurysm was selectively cannulated using a coaxial technique (5f Sim I catheter; Cordis Corporation, Bridgewater, NJ, USA, and 2.7f Progreat micro-catheter; Terumo Corp, Tokyo, Japan) and embolized (5 mm and 7 mm Concerto™ Helix; Medtronic Inc, Santa Rosa, CA, USA, and 7mm Ruby™ coils; Penumbra, Alameda, CA, USA) after superselective angiography (Figure 1). Procedural success was immediately achieved as confirmed by completion angiography (Figure 2). Femoral access was closed using a FemoSeal^®^ device (Saint Jude, St. Paul, MN, USA) implantation.

**Figure 3:**
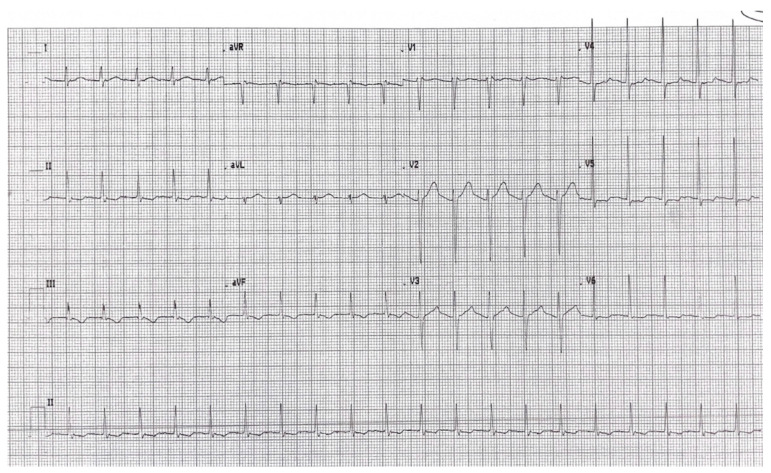
Postoperative electrocardiography showing supraventricular tachycardia and signs of left ventricle overload.

The postoperative course was uneventful, until the fifth postoperative day when the patient experienced a sudden onset of tachycardia and oppressive thoracic chest pain. Electrocardiography examination showed a supraventricular tachycardia with a heart rate of 120 beats/minute bpm and signs of left ventricle overload (Figure 3). The patient was unresponsive to a Valsalva maneuver and carotid sinus massage. 

A subsequent transthoracic echocardiogram showed no significant pathologic findings; the left ventricle was normal for size and thickness, maintained ejection fraction (68%), showed no pulmonary artery hypertension, and only a mild mitral valve insufficiency. Therefore, the patient was medically managed by 4.000 IU low-molecular-weight heparin per day, associated with intravenous administration of amiodarone lasting for 72 h with complete symptom regression. Once the normal sinus rhythm was reestablished, oral medication with 400 mg/day of amiodarone was continued for the following five days. 

Prior to hospital discharge, a new CTA showed complete splenic artery aneurysm exclusion and a non-surgical polar splenic infarction (Figure 2). Blood samples were negative, except for mild leukocytosis (18.44 × 10^3^/μL) and thrombocytosis (602 × 10^3^/μL).

The patient was discharged in good general condition on the 14th postoperative day under single antiplatelet (acetylsalicylic acid 100 mg/die) and anti-hypertensive (verapamil 80 mg twice/daily) drugs. At 1 year of follow-up, he was still completely asymptomatic with no recurrent symptoms.

## 3. Discussion

Actins, as is already known, constitute a family of highly conserved cytoskeletal proteins that are indispensable for cellular function. All vertebrates encode six tissue-specific actin isoforms: two in striated-muscle cells (skeletal and cardiac), two in smooth-muscle cells (SMCs) (vascular and visceral), and two in non-muscle cells [2,3]. Among them, vascular actin is the most represented protein in SMCs (40% of the total protein amount, 70% of the total actins) [10,11,12]. 

The first evidence that mutations affecting an SMC contractile protein cause familial vascular disease was associated with the identification of MYH11 mutations leading to thoracic aortic dissections and aneurysmatic degeneration in association with patent ductus arteriosus [13,14].

Regarding actin codifying genes’ mutations, their molecular consequences can be explained by the structure of actin itself. Any mutation resulting in alteration of the chemical structural makeup of residues that comprise this vital binding region are expected to be deleterious. Indeed, the major function of vascular smooth muscle cells (SMCs) is to contract in response to the stretch resulting from pulsatile blood flow, a process that is dependent on the cyclic interaction between thin filaments, composed of the SMC-specific isoform of α-actin (SM α-actin, encoded by ACTA2), and thick filaments, composed of SMC-specific β-myosin [9,15].

The c*.971G > A p.Arg324Gln* variant is extremely rare with a reported allele frequency of 0.0000398. It is well known that ACTN2 gene mutation patients are more prone than the general population to developing heart disease, stroke occurrence, and aortic aneurysm [9]. A possible explanation is related to the intrinsic α-actin role in regulating the cardiac expression of sodium channels on the cell membrane by binding to its spectrin-like domains [16]. In the case of mutation, spectrin-like binding sites could be altered, contributing to the dysregulation of ion channels and inducing aberrant cardiac action potentials causing cardiac arrhythmias [17,18,19,20,21]. 

Notably, according to Gou and co-workers [9], α-actin mutations lead to the dilatation of large arteries, like the thoracic aorta, and the occlusion of medium/smaller arteries. 

This differential response to a single underlying defect in SM α-actin may be due to several factors:

1. The aorta is an elastic artery, in which the media is composed of SMCs lying between layers of elastic fibers. In contrast, medium and small arteries are muscular arteries, in which the SMCs are attached to each other and not the layers of elastic fibers [22]. Moreover, elastin has an established role in increasing SMC differentiation and decreasing SMC proliferation, and the lack of SMC and elastic fiber interaction may increase proliferation in muscular arteries [23].
2. The aorta (especially its thoracic portion) bears most of the pulsatile blood forces, whereas other arteries experience significantly less stress. Those physiologically different forces may activate different pathways in the mutant SMCs, leading to different vascular disease presentation [24].

Nevertheless, our unusual case seems to contradict this clear established dichotomy between large and medium/small arterial disease in patients with α-actin mutation. 

Although several genetic disorders (especially, but not limited to, Ehlers–Danlos syndrome) are related to splenic artery dilatation and even true aneurysmal degeneration [25,26,27,28], to the best of our knowledge, this case shows, for the first time, the occurrence of the *c.971G > A p.Arg324Gln* missense variant ACTN2 gene linked to splenic aneurysm occurrence in a healthy young man. 

Speculatively, we could hypothesize a possible correlation between ACTN2 mutations and aneurysm development in different arterial vessels (like visceral ones) rather than only in the aorta, as previously suggested [9]. 

## 4. Conclusions

Although *c.971G > A p.Arg324Gln* missense mutation is an extremely rare occurrence in the general population, there is undebatable proof of a correlation between ACTN2 gene mutation and cardiovascular disease occurrence in otherwise young and healthy subjects. 

Nowadays, those patients are routinely included in cardiological screening and follow-up programs. Our observation could suggest the necessity to perform a complete peripheral vascular artery assessment, as well as a cardiological evaluation.

Undoubtedly, further studies are needed to confirm our hypothesis of a potential correlation between ACTN2 gene mutation and the development of visceral aneurysms.

## Data Availability

The data presented in this study are available on request from the corresponding author. The data are not publicly available due to their containing information that could compromise the privacy of research participants.
